# iS-CellR: a user-friendly tool for analyzing and visualizing single-cell RNA sequencing data

**DOI:** 10.1093/bioinformatics/bty517

**Published:** 2018-07-04

**Authors:** Mitulkumar V Patel

**Affiliations:** Immunocore Ltd, Abingdon, Oxfordshire, UK

## Abstract

**Summary:**

Interactive platform for single-cell RNA-sequencing (iS-CellR) is a web-based Shiny application that is designed to provide user-friendly, comprehensive analysis of single-cell RNA sequencing data. iS-CellR has the capability to run on any modern web browser and provides an accessible graphical user interface that enables the user to perform complex single-cell RNA-sequencing analysis without requiring programming skills.

**Availability and implementation:**

iS-CellR is open source and available through GitHub at https://github.com/immcore/iS-CellR. iS-CellR is implemented in Docker and can be launched on any operating system with Docker installed.

**Supplementary information:**

Supplementary data are available at *Bioinformatics* online.

## 1 Introduction

Interactive platform for single-cell RNA-sequencing (iS-CellR) enables the high-throughput quantification of gene expression profiles of individual cells and the discovery of cellular heterogeneity and functional diversity (Shapiro *et al.*, 2013). The increased complexity of single-cell RNA-sequencing (scRNA-seq) data presents significant challenges for the effective analysis and interpretation of results. Numerous pipelines and statistical methods have been developed for scRNA-seq data including Model-based Analysis of Single-cell Transcriptomics (MAST) (Finak *et al.*, 2015), single cell differential expression (SCDE) (Kharchenko *et al.*, 2014), single-cell consensus clustering (SC3) (Kiselev *et al.*, 2017), Seurat (Satija *et al.*, 2015), SINgle Cell RNA-seq profiling Analysis (SINCERA) (Guo *et al.*, 2015) and reference component analysis (RCA) (Li *et al.*, 2017). However, all of these pipelines and methods are embedded in R language and require R programming skills. In response, several graphical user interface/web-based applications have been developed for scRNA-seq data analysis including Automated Single-cell Analysis Pipeline (ASAP) (Gardeux *et al.*, 2017), Ginkgo (Garvin *et al.*, 2015), SCell (Diaz *et al.*, 2016) and FastProject (DeTomaso and Yosef, 2016). However, most of these stand-alone applications have dependencies and require installation of specific packages. In addition, these applications do not offer a comprehensive analysis of single-cell data, have a rigid workflow and do not offer essential features such as quantitatively comparing heterogeneities within and/or between cell populations, and saving, sharing and reproducing results over time.

Single-cell RNAseq Analysis Pipeline, iS-CellR, was developed to provide a comprehensive analysis of scRNA-seq data, using an open-source R-based platform with a user-friendly graphical interface. iS-CellR integrates Seurat package and employs a fully integrated web browser interface to process, analyse and visually interpret scRNA-seq data. This single web-based platform can be utilised by a full spectrum of researchers, from biologists to computational scientists, to study cellular heterogeneity.

## 2 iS-CellR platform

iS-CellR is open source and available through GitHub at https://github.com/immcore/iS-CellR. iS-CellR is written using the R programming language, and is built with the Shiny framework (R Studio Inc, 2013). iS-CellR can be launched locally using any R environment including RStudio, R Console, etc. In addition, to encourage reproducibility and to make the programme platform independent, iS-CellR is also wrapped into Docker (Merkel, 2014). All the dependencies of iS-CellR are included in the Dockerfile, and iS-CellR can be launched with the single Docker run. Upon launching iS-CellR with or without Docker, all the required dependencies of iS-CellR will be checked and installed seamlessly without any user input. The front-end of iS-CellR dynamically loads the graphical components and provides a full user-friendly interface using ShinyJS (https://cran.r-project.org/package=shinyjs). iS-CellR allows a complete workflow analysis to be completed in minutes by leveraging Shiny’s reactive framework, which enables the compartmentalization and cache of essential but expensive pipeline steps to avoid unnecessary recomputations during each session.

The current implementation of iS-CellR provides wrapper functions for running the Seurat package for scRNA-seq data and translates user-driven events (e.g. button clicks and checkbox) into R reactive objects, and display interactive results as dynamic web content. iS-CellR incorporates five key features in a single platform for in-depth analysis of scRNA-seq data and assists the user with interactive analysis and sophisticated visualization:

1. iS-CellR integrates R packages via wrapping with Shiny user-interface elements and rendering the resulting plots. iS-CellR completely replaces the commands and lines of code for many packages with buttons, checkboxes and other graphical controls, and displays results using an interactive plotting environment with controls such as zoom in and out, selecting and highlighting data points, scaling axes and mouse hover information.
2. iS-CellR allows visualization of co-expressed genes simultaneously. This feature is helpful when comparing the expression levels of two genes in response to drug treatment. This attribute allows the user to enter the names of two genes and choose the expression threshold to visualize their relative expression simultaneously.
3. iS-CellR can quantify cellular heterogeneity based on pre-selected sets of marker genes, considering heterogeneity within and/or between samples. Average expression signatures for two different gene sets define transcriptional cell states of each sample. The associated expression of both gene sets may vary between samples and their relative correlation across individual cells within samples may have its own biological significance.
4. iS-CellR uses Shiny’s reactive programming framework to allow the results generated by one step to be used in subsequent steps, avoiding recomputations and enabling faster running.
5. iS-CellR offers the ability to reproduce exactly the same results and allow the sharing of data over time. It provides the user a single button click at the end of the analysis to generate a HTML report automatically. It also allows the user to download publication-quality plots.

## 3 Example

**Fig. 1.**
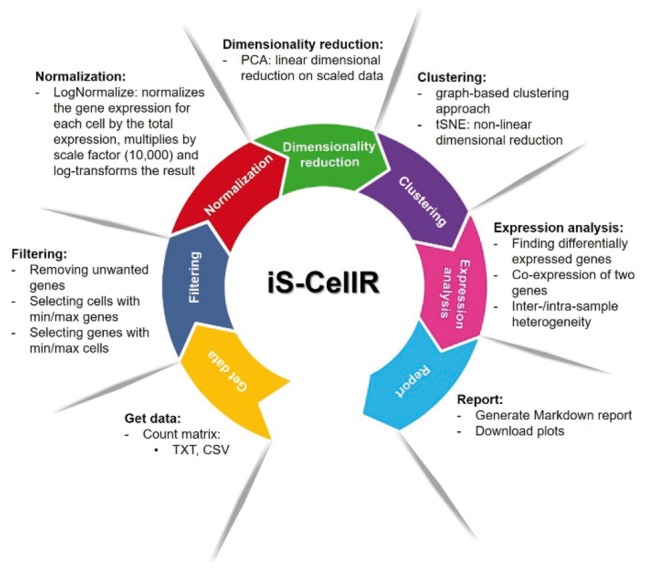
iS-CellR pipeline overview. iS-CellR is organized into a seven-step process for complete scRNA-seq analysis. The user can interactively select steps to perform analysis using single-cell data. After uploading, the raw data are filtered and normalized. The normalized data are then subjected to dimensionality reduction for principle component analysis (PCA). Further dimensionality reduction can be performed using t-distributed stochastic neighbour embedding (tSNE). After a clustering step, differentially expressed marker genes can be visualized on cell clusters. The user can also visualize co-expression of two genes simultaneously. Inter-/intra-sample heterogeneity requires the user to upload a file with a list of genes in a two-column format (GeneSet1, GeneSet2). Finally, the user can generate a HTML report containing all results produced or download plots individually

iS-CellR was applied to a published data set (Tirosh *et al.*, 2016), which analysed scRNA-seq data from 4645 single cells (isolated from 19 patients) to study distinct genotypic and phenotypic states of melanoma tumours. iS-CellR produced similar findings to the published study, handling thousands of samples on a single platform in minutes (see demonstration with published melanoma scRNA-seq data in Supplementary Material). A typical workflow in iS-CellR is summarized in Figure 1 and further detailed in Supplementary Material.

## 4 Conclusion

iS-CellR offers a comprehensive strategy for the analysis and visualization of scRNA-seq data without the need for specific programming skills. iS-CellR allows researchers to quickly and easily explore heterogeneous populations of cells. iS-CellR can be modified and extended according to user needs to perform more intricate and targeted analysis. Future work on iS-CellR will include incorporating established methods to the platform (e.g. SC3 and RCA), extending analysis workflow with pseudotime analysis, and providing hyperlinks to external databases for detailed annotation.

## Funding

This work was supported by Immunocore Ltd.

*Conflict of Interest*: Mitulkumar Patel is an employee of Immunocore Ltd.

## Supplementary Material

Supplementary Information
