# Supplementary material for: iS-CellR: a user-friendly tool for analyzing and visualizing single-cell RNA sequencing data
Source: Bioinformatics. 2018 Jul 4;34(24):4305–6. doi: 10.1093/bioinformatics/bty517 (PMC6289135; doi:10.1093/bioinformatics/bty517)
Supplement: Supplementary Information [file bty517_supplementary_information.docx]

**Supplementary Information for:**

**iS-CellR: a user-friendly tool for analysing and visualizing single-cell RNA sequencing data**

Mitulkumar V. Patel*^1^

^1^Immunocore Ltd, Oxfordshire, OX14 4RY, UK

*Corresponding author

**iS-CellR: demonstration with published melanoma single-cell RNA sequencing data**

Here, the features of iS-CellR (Interactive platform for single-cell RNA-sequencing) are demonstrated using a published dataset for metastatic melanoma (Tirosh *et al.,* 2016). In their study, Tirosh and colleagues sampled single-cell RNA-sequencing (scRNA-seq) data from 4,645 single cells (isolated from 19 patients) to investigate distinct genotypic and phenotypic states of melanoma tumours (Tirosh *et al.,* 2016). For demonstration purposes described herein, only the published malignant cell dataset has been selected; the raw data are available with the published paper (Tirosh *et al*., 2016).

**Prerequisite**

1). Install Docker (v >= 18.02.0-ce)

Download and install Docker from <https://docs.docker.com/install/>

2). Install R (v >= 3.2)

Download and install R from <http://cran.us.r-project.org/>

3). Install R studio (Optional)

Download and install RStudio Desktop from <http://rstudio.org/download/desktop>

4). Install Shiny package

install.packages(“shiny”) # inside R console

**Launch iS-CellR**

There are four different ways to launch iS-CellR:

# Option 1: the easiest way is to use Docker image.

docker run -p 3838:3838 immcore/is-cellr # on command line

# Option 2: using Url.

runUrl('https://github.com/immcore/iS-CellR/archive/master.zip') # inside R console

# Option 3: using runGitHub.

shiny::runGitHub("iS-CellR", "immcore") # inside R console

# Option 4: clone the git repository, then use runApp().

runApp(/fullpath/iS-CellR) # inside R console

**Troubleshooting**

Seurat installation

When you see the following warning message in the R console:

Warning: Installed Rcpp (0.12.12) different from Rcpp used to build dplyr (0.12.11).

Please reinstall dplyr to avoid random crashes or undefined behavior.

install.packages("dplyr", type = "source")

library(dplyr)

If you are using older R versions, installing from source causes errors on some standard Mac installs due to R being compiled using gfortran-4.8. The error will look something like

-L/usr/local/lib/gcc/x86_64-apple-darwin13.0.0/4.8.2'

ld: library not found for -lgfortran

To fix the above error type following commands in terminal. Sudo permissions required

curl -O http://r.research.att.com/libs/gfortran-4.8.2-darwin13.tar.bz2

sudo tar fvxz gfortran-4.8.2-darwin13.tar.bz2 -C /

**iS-CellR: homepage**

After launching iS-CellR, the user will be able to view the homepage in the web browser (Figure S1).


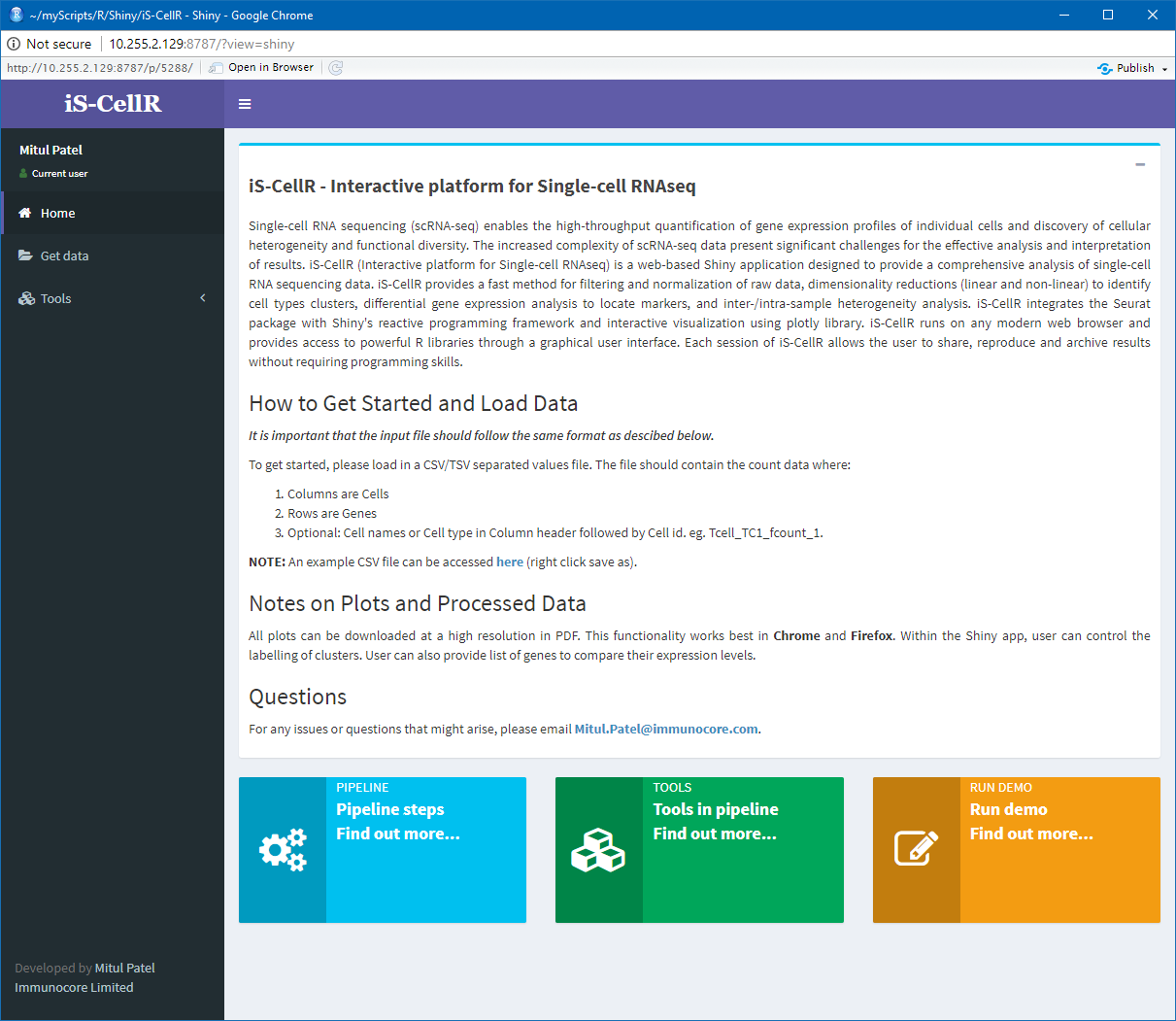


**Fig. S1. Homepage.** The iS-CellR homepage introduces scRNA-seq analysis along with guidelines and requirements for using iS-CellR.

**iS-CellR: uploading data**

To run iS-CellR, the first step is to upload matrix data. The matrix should be in a TXT/CSV file, with columns representing cell types and rows representing genes. Column headers can be used to assign sample or cell type to the clusters. Each column header must start with the sample name (or cell name) followed by “_” or “.”. e.g. sample1_sc1_123 or sample1.sc1_123. After uploading the file, the user can view the file contents in the Data panel (Figure S2). The Data panel also allows the user to perform searches and sort columns. The user is then able to run iS-CellR via the Tools>iS-CellR sub-menu option.


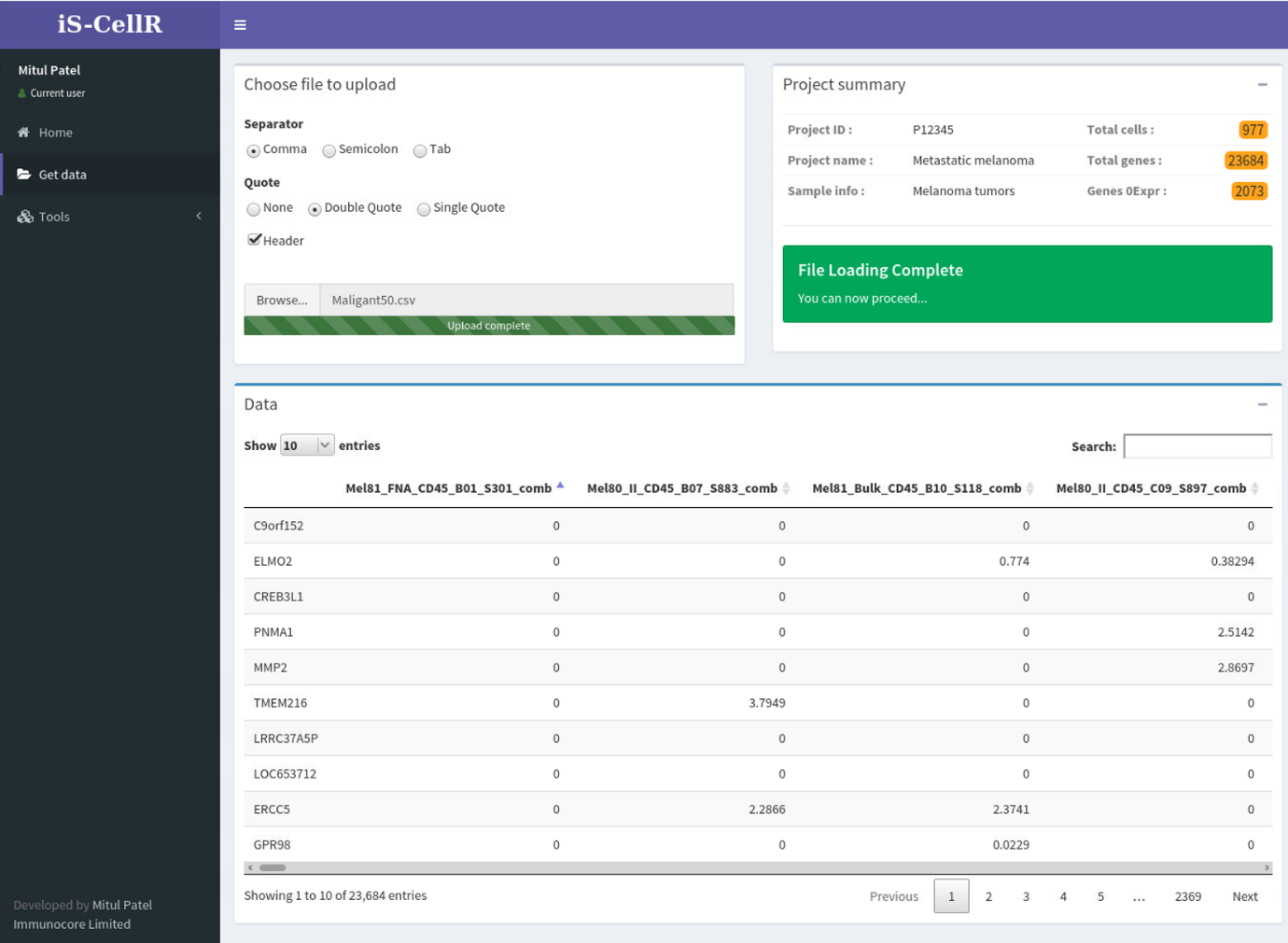


**Fig. S2. Loading data.** The user can upload a matrix file in specified format and view the data in the data table underneath. iS-CellR provides a summary of the data on right-hand side including the number of cells and genes, and the percentage of genes with no (zero) expression. The status flag turns from red to green to confirm that data have been uploaded successfully and the user can proceed to run the pipeline.

**iS-CellR: running pipeline**

iS-CellR offers the following six steps to explore the dataset (Figure S3): 1). quality control and cell filtering, 2). detecting variable genes 3). linear dimensionality reduction (principle component analysis (PCA)), 4). non-linear dimensionality reduction (t-distributed stochastic neighbour embedding (tSNE)), 5). identifying differentially expressed genes and 6). discriminating markers (studying inter-/intra-sample heterogeneity and visualising co-expression of two genes simultaneously). The user has the choice to select individual or multiple steps to run the pipeline. iS-CellR also allows the user to switch between steps during each running session. iS-CellR will then produce panels for the selected steps and allows the user to interpret the results from interactive plotting. iS-CellR provides full user-friendly support via events such as enable/disable and show/hide actions on graphical elements.


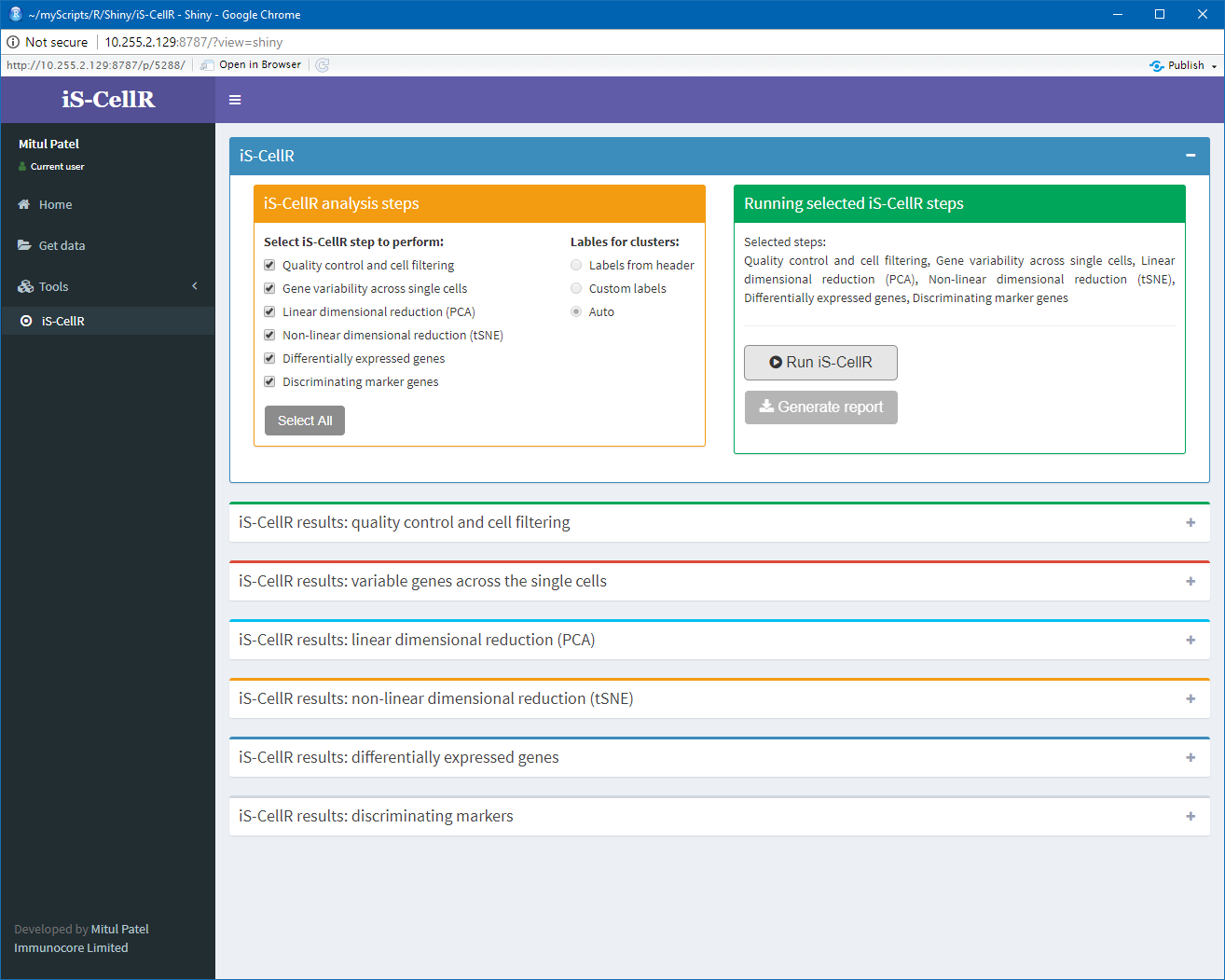


**Fig. S3. Running iS-CellR.** After uploading data, the user can select the desired ‘iS-CellR analysis steps’ for analysis and perform the initial iS-CellR run. The disabled button ‘Run iS-CellR’ will automatically be enabled after uploading data and selecting steps. The resulting panels will be generated for each selected step.

Cluster annotation feature (“Labels for clusters”) under iS-CellR analysis step (Figure S3) allows user to annotate or name the clusters in three different ways. 1). cluster numbers (0,1…n, default) 2). custom names or 3). column headers. User can switch to any option from any tSNE/PCA plot and the effect will be applied to all remaining plots. Clusters will be annotated by numbers as a default setting. User can add custom names for each of the numbered cluster using the excel style input window. If selected, the cell name will be extracted from column headers and used to annotate clusters.

**iS-CellR: quality control and cell filtering**

The panel for quality control and cell filtering will allow the user to perform QC analysis on the expression matrix. The summary plot showing frequency of expression (across all genes) vs mean expression (of all genes) will be rendered automatically (Figure S4). The user can also query the plot using the number of genes and expression values to extract more information. iS-CellR allows the user to apply or remove filtering at any stage of the analysis and the relevant effect will be automatically applied to certain completed steps while the others requires plot button to be clicked. The user can apply or remove filters based on three thresholds, including minimum cells (genes must be in a minimum number of cells), minimum genes (cells must have at least the minimum number of genes) and minimum expression value. The default filter is minimum cells:3, minimum genes:3 and expression value:0.

**
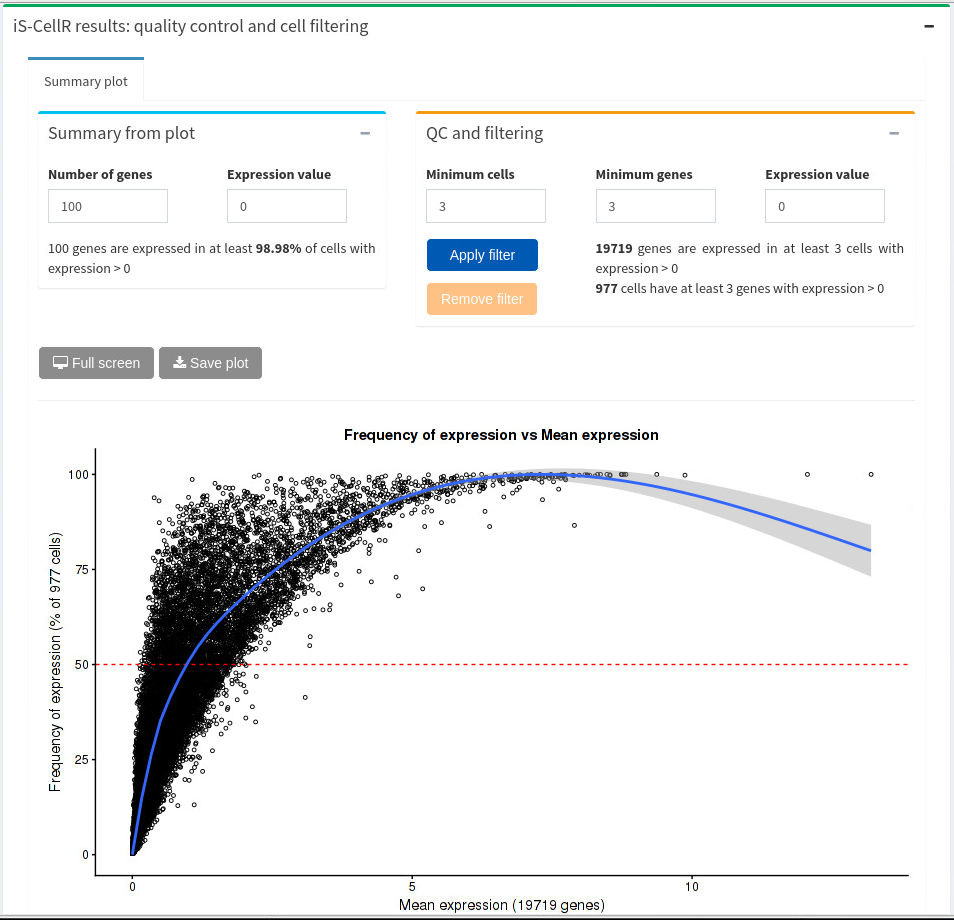
Fig. S4. QC analysis.** The scatterplot shows the frequency of genes expressed across cells against the mean expression of those genes. The user can specify the filter option to apply the filter on the dataset by clicking the relevant button. The ‘Remove filter’ button will remove any filters applied and set back to the default filter. The panel also provides useful information while specifying the filter values prior to applying filter, which guides user to select the desired filter.

**iS-CellR: detecting variable genes**

The panel for variability in gene expression (Figure S5) will be generated automatically after selecting the relevant option following the initial iS-CellR run. This collapsible panel contains one tab, which displays a scatter plot of the average expression and dispersion for each gene. This provides information on heterogeneity in the sample and labels any outliers.


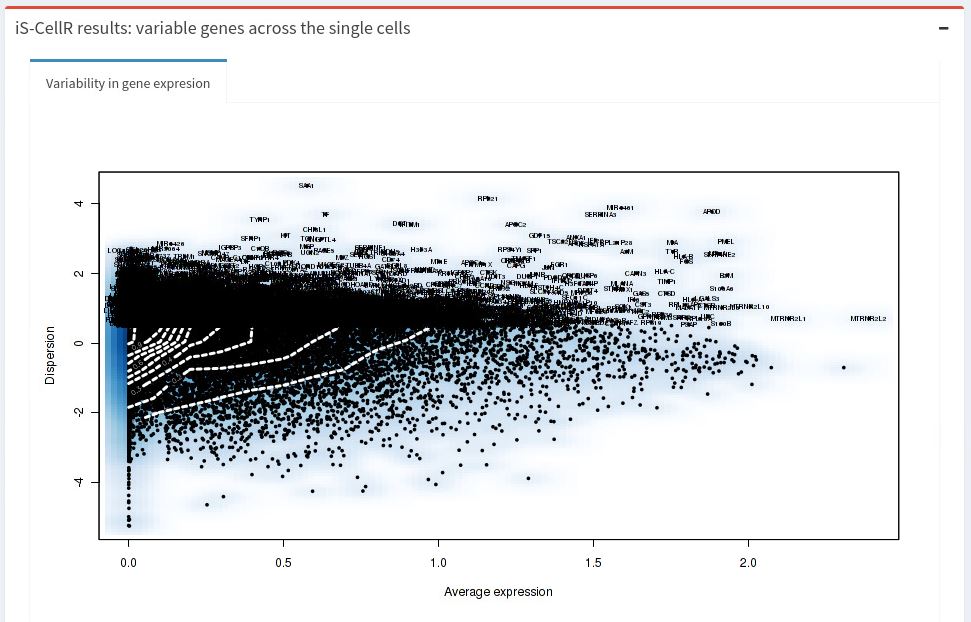


**Fig. S5. Variable genes.** A smooth scatter plot with mean variance relation.

**iS-CellR: linear dimensionality reduction, PCA**

The panel for linear dimensionality reduction will be generated automatically after selecting the relevant option and running initial iS-CellR steps. The panel is collapsible and contains four tabs including PCA plot in 2-dimensions (2D) (Figure S6A); PCA plot in 3D (Figure S6B); visualizing PCA (Figure S6C); and PCA Heatmap (Figure S6D). The first tab displays the PCA plot in 2D and allows the user to add label annotations to the plot by selecting a checkbox. The PCA plot is interactive and offers a range of features such as zooming in and out, highlighting data points, rescaling axes, mouse hover information and the ability to download the plot. PCA plots in 2D use PC1 and PC2, while plots in 3D additionally use PC3.


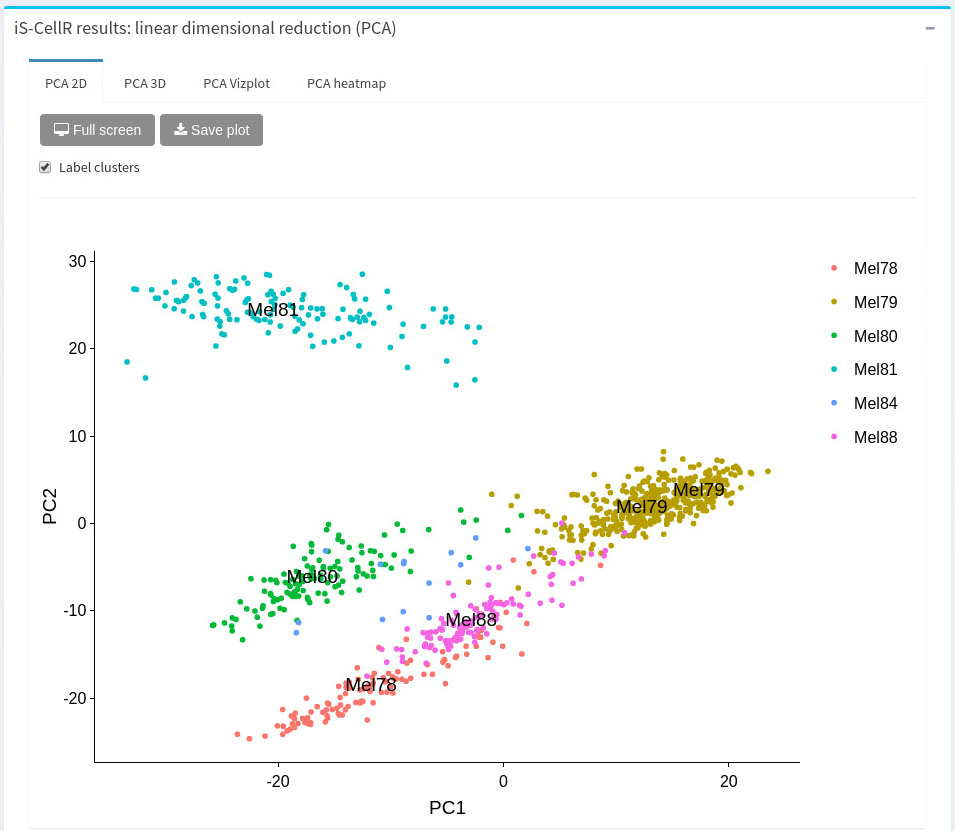


**Fig. S6A. Linear dimensionality reduction, PCA 2D.** PCA of the scRNA-seq data of six tumour types, including Mel78, Mel79, Mel80, Mel81, Mel84 and Mel88. The first (PC1) and second (PC2) components of the PCA are shown on each axes, where each point corresponds to a sample and each colour corresponds to a tumour type.


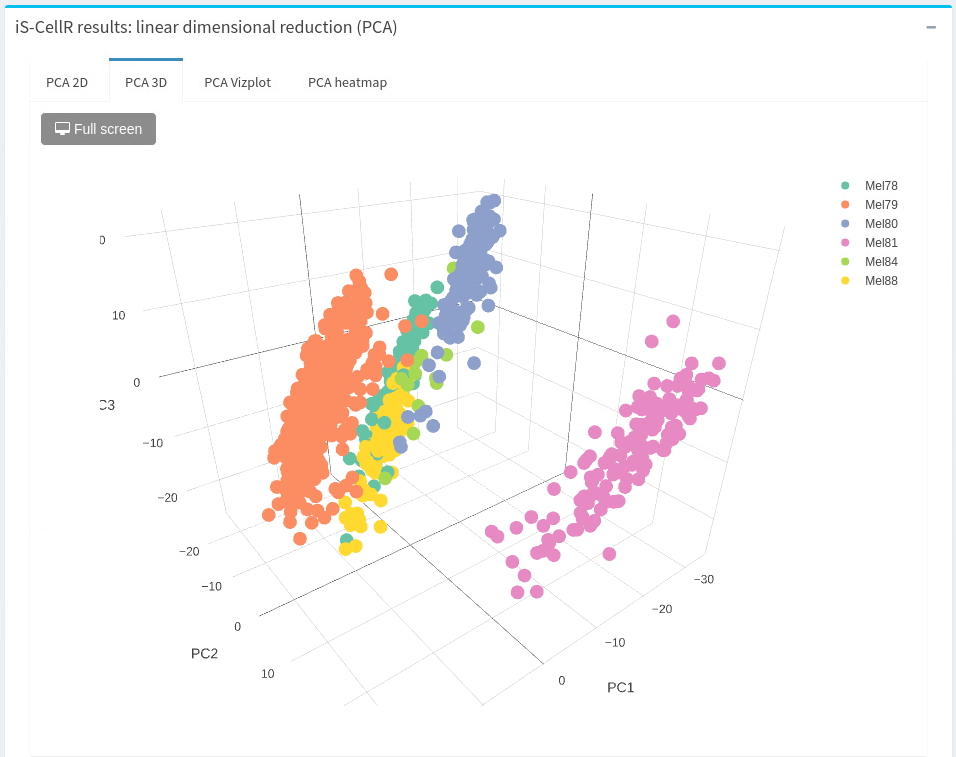


**Fig. S6B. Linear dimensionality reduction, PCA 3D.** PCA plotted in 3D from the same six tumour types as shown in Figure S6A. The plot is projected using the first three components (PC1, PC2 and PC3) from the PCA analysis.

**
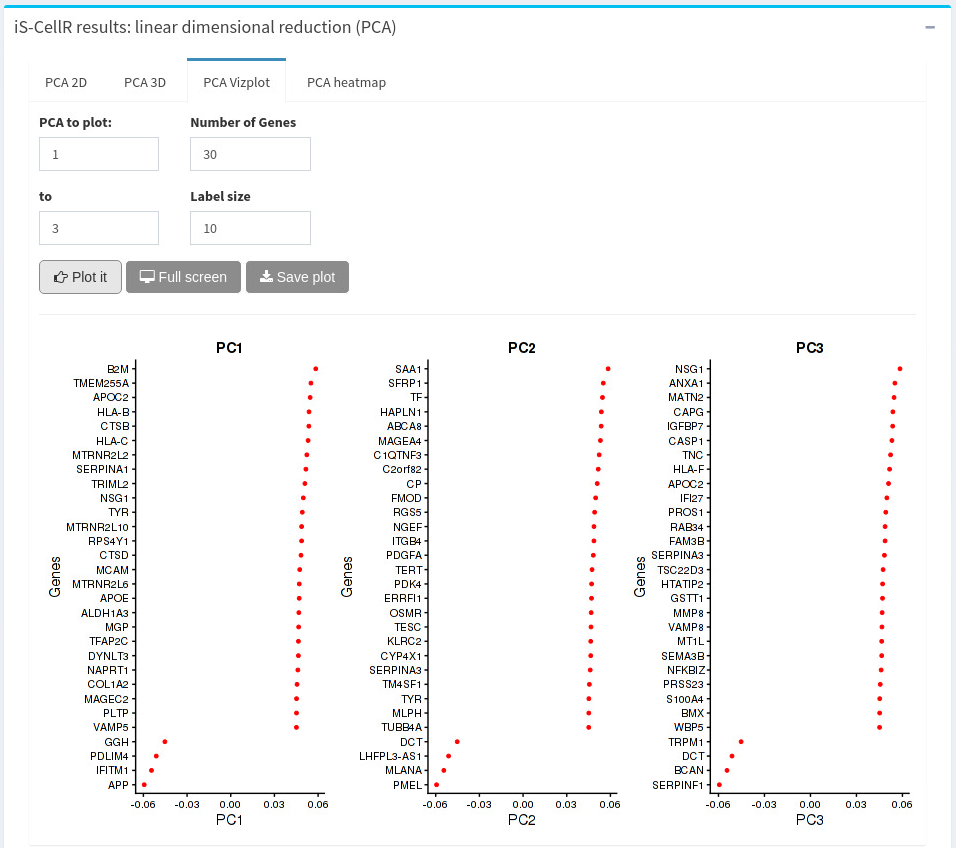
**

**Fig. S6C. Linear dimensionality reduction, PCA visualization.** The top 30 genes are ranked by absolute score values associated with reduction components. The user can specify the number of genes and reduction components to use, as well as font size of axis labels.


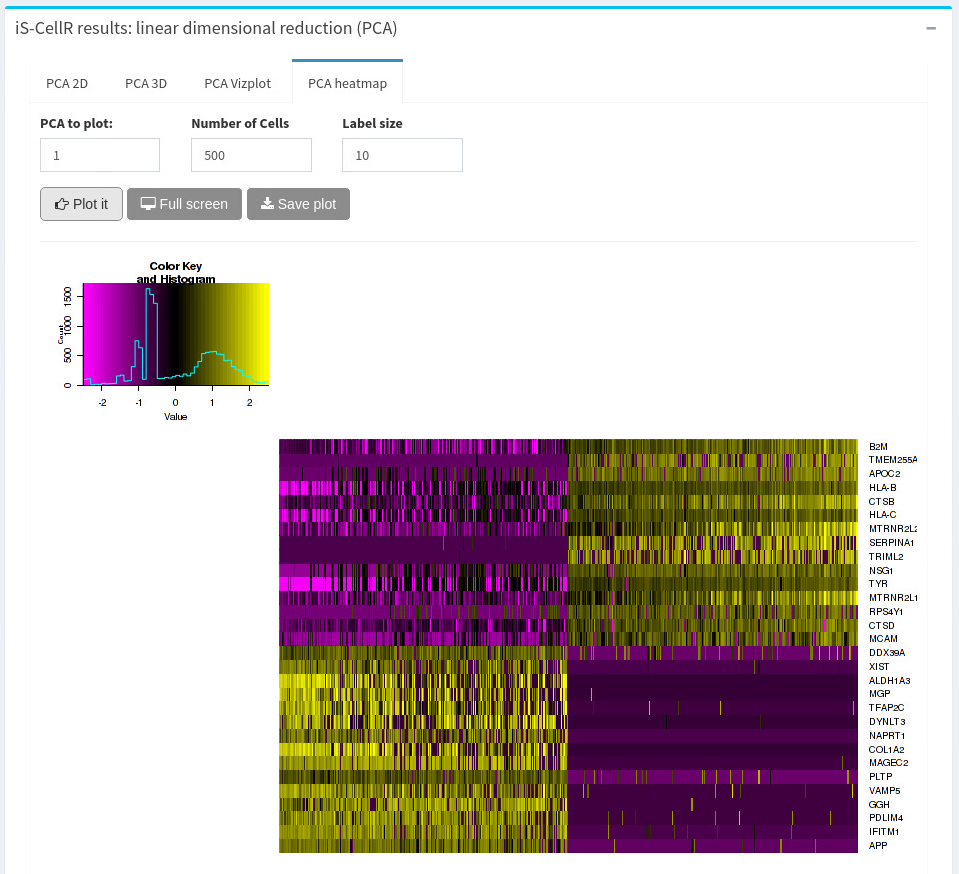


**Fig. S6D. Linear dimensionality reduction, PCA Heatmap.** The PCA heatmap is produced using the first principle component (PC1) scores for 30 genes across 500 cells. Both genes and cells are ordered according to their PCA scores. The user can specify parameters to fulfil the requirements. The heatmap offers easy exploration of the cellular heterogeneity within the dataset and also guides the user to identify the optimal PCs for further downstream analysis.

**iS-CellR: non-linear dimensionality reduction, tSNE**

The panel for non-linear dimensionality reduction will be generated automatically after selecting the relevant option and running initial iS-CellR steps. The panel is collapsible and contains four tabs to display tSNE plots in 2D and 3D. The first and second tabs display the tSNE plots in 2D (Figure S7A) and 3D (Figure S7B), respectively, with similar interactive features as PCA plots. The tSNE plot on the third tab (tSNE select) allows the user to select data points (by dragging the mouse) on the plot and relative information of selected points will be presented in the table next to the plot (Figure S7C). The tSNE plot on the fourth tab (tSNE highlight) uses data (subset of data) presented in the table next to the plot to highlight data points on the plot (Figure S7D). The user can also search the data table (via search box) with the cell name, cluster id or sample name and the relative points will be highlighted automatically on the plot. Both of these tabs the ease of interpreting high dimensional data with simple clicks and selection.


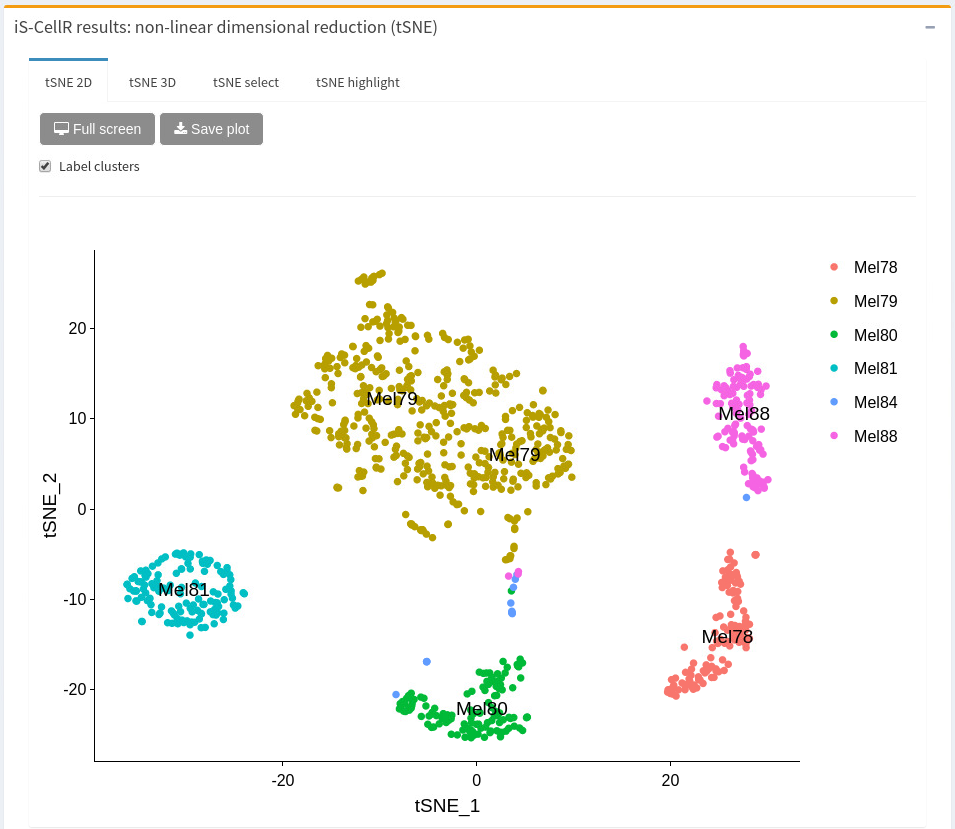


**Fig. S7A. Non-linear dimensionality reduction, tSNE 2D.** A tSNE plot of six malignant tumours projected onto the first two tSNEs. Clusters are labelled and coloured according to the tumour type and the cells from the same tumour type are clustered together, as expected. The user can remove cluster labels by unchecking the checkbox in the top left-hand corner.


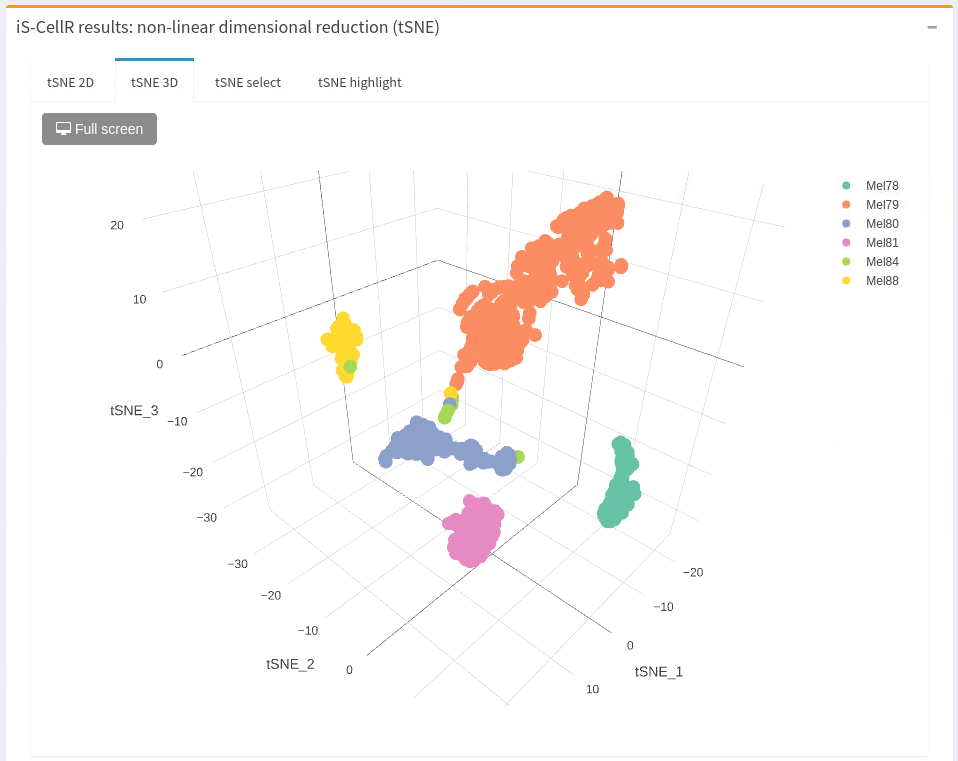


**Fig. S7B. Non-linear dimensionality reduction, tSNE 3D.** A tSNE plot in 3D for the same six tumour types as shown in Figure S7A. The plot uses the first three tSNEs such as tSNE1, tSNE2 and tSNE3. The user can expand the view by selecting the ‘Full screen’ button.


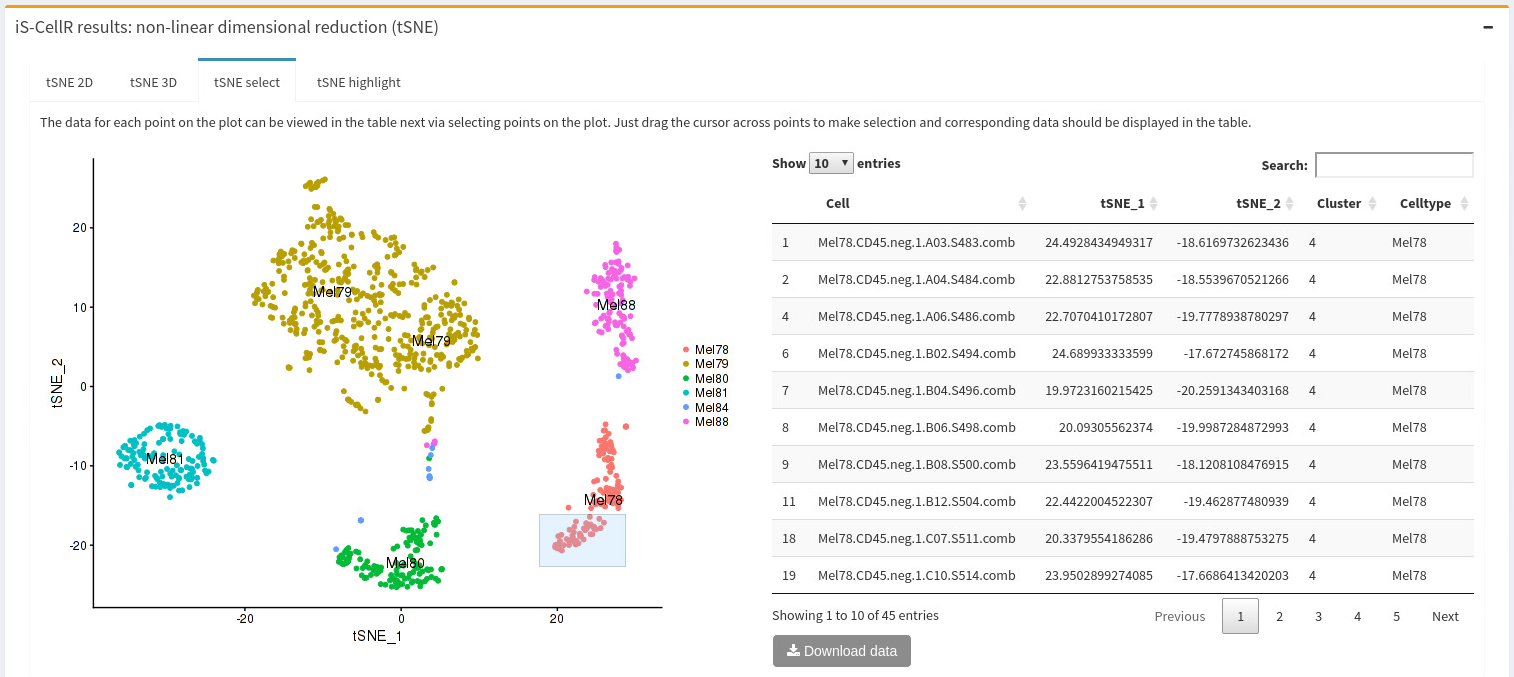


**Fig. S7C. Non-linear dimensionality reduction, tSNE select.** tSNE select allows the user to view data corresponding to clusters by selecting one or more data points. Data from the selected points are rendered into the data table to provide further information on specific clusters or groups of cells of interest.


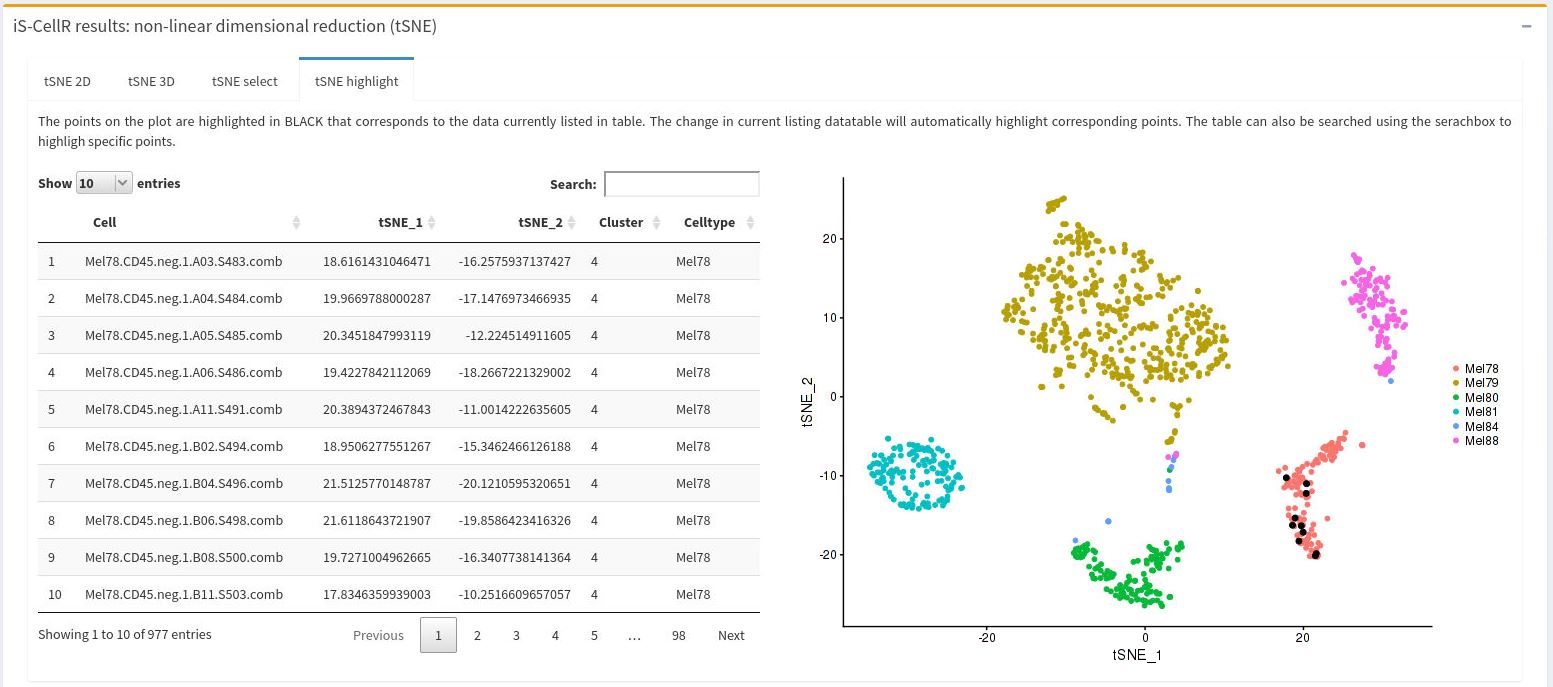


**Fig. S7D. Non-linear dimensionality reduction, tSNE highlight.** tSNE highlight allows the user to query the plot. The count matrix is loaded into the data table and the currently listed cells are highlighted in black in the tSNE plot on right-hand side. The tSNE plot will automatically update to reflect the changes in the data table. The user can also search the data table for specific cell names using the search box.

**iS-CellR: identifying differentially expressed genes**

The panel for differentially expressed genes will automatically be generated after selecting the relevant step and following the initial iS-CellR run. This panel allows the user to identify and locate differentially expressed genes or marker genes across clusters of cells. The panel offers five different ways to visualise, including a Joy plot (Figure S8A), Violin plot (Figure S8B), Dot plot (Figure S8C), Scatter plot (Figure S8D), and Heatmap (Figure S8E) to study differentially expressed genes. Each tab allows the user to enter a list of gene names separated by a comma (“,”) in the textbox and subsequently generates the plot. Using these plots the user can easily identify and locate genes of interest across the clusters with relative expression levels. The full screen button on each tab allows the user to expand the plot for better visualization.


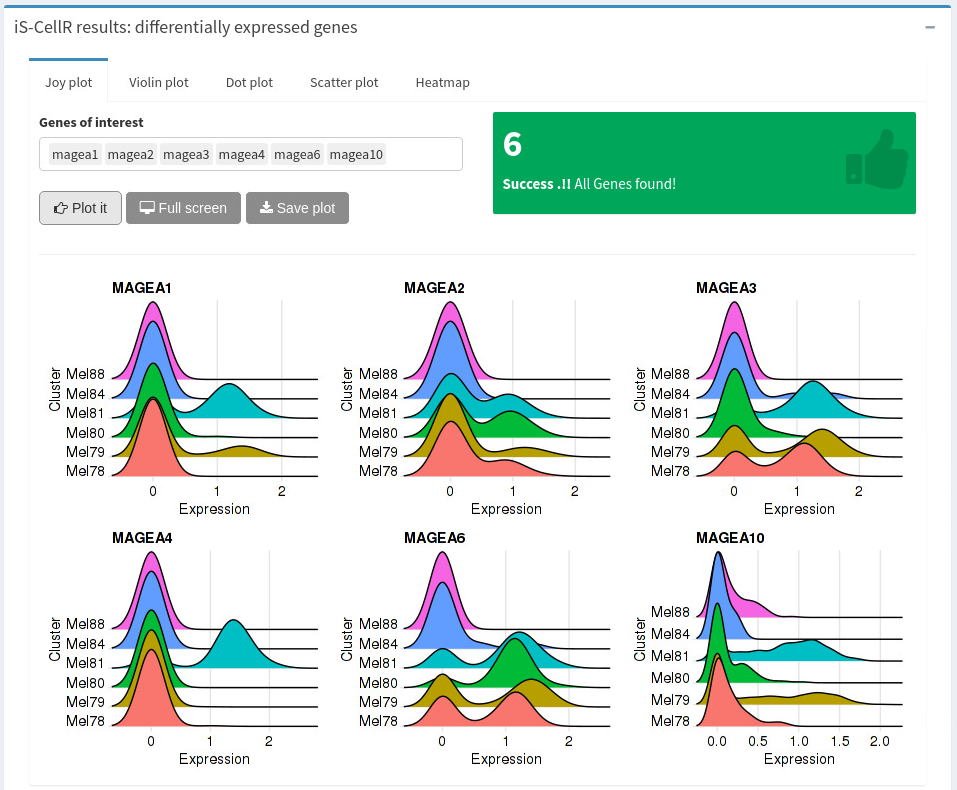


**Fig. S8A. Differentially expressed genes – Joy plot.**


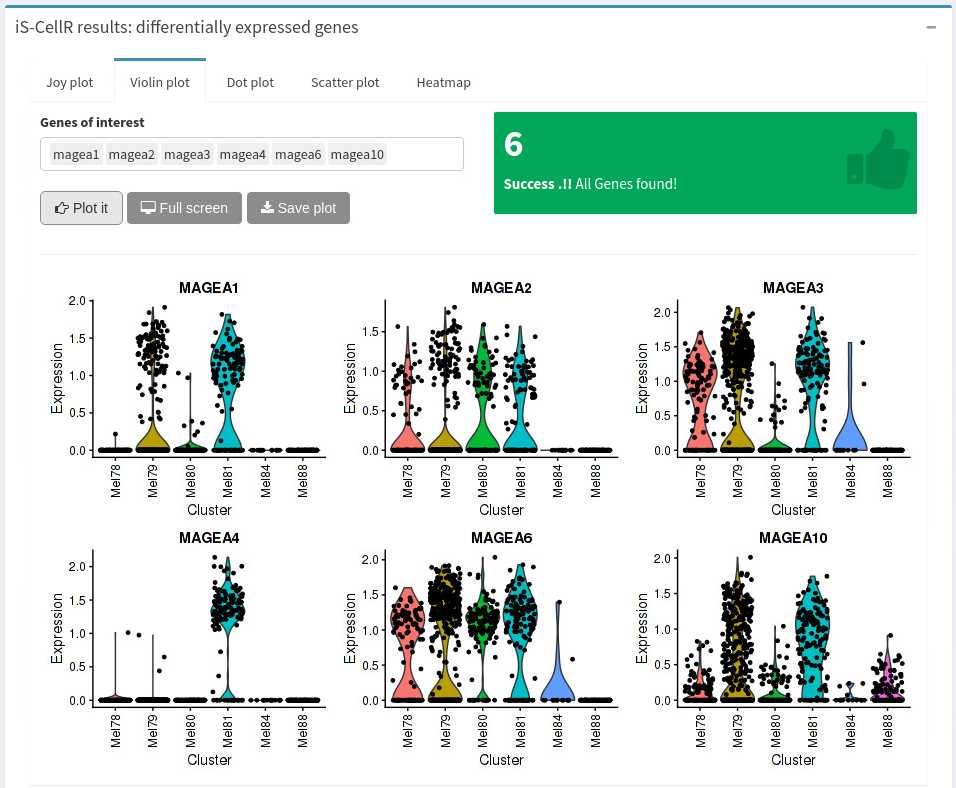


**Fig. S8B. Differentially expressed genes – Violin plot.**


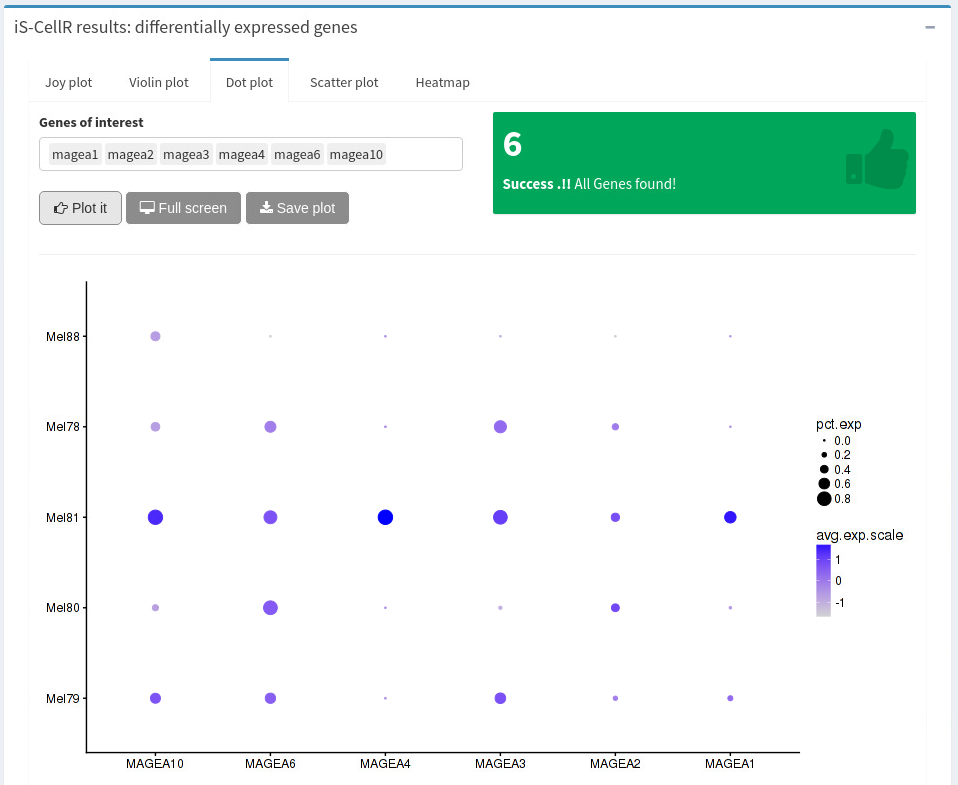


**Fig. S8C. Differentially expressed genes – Dot plot.**


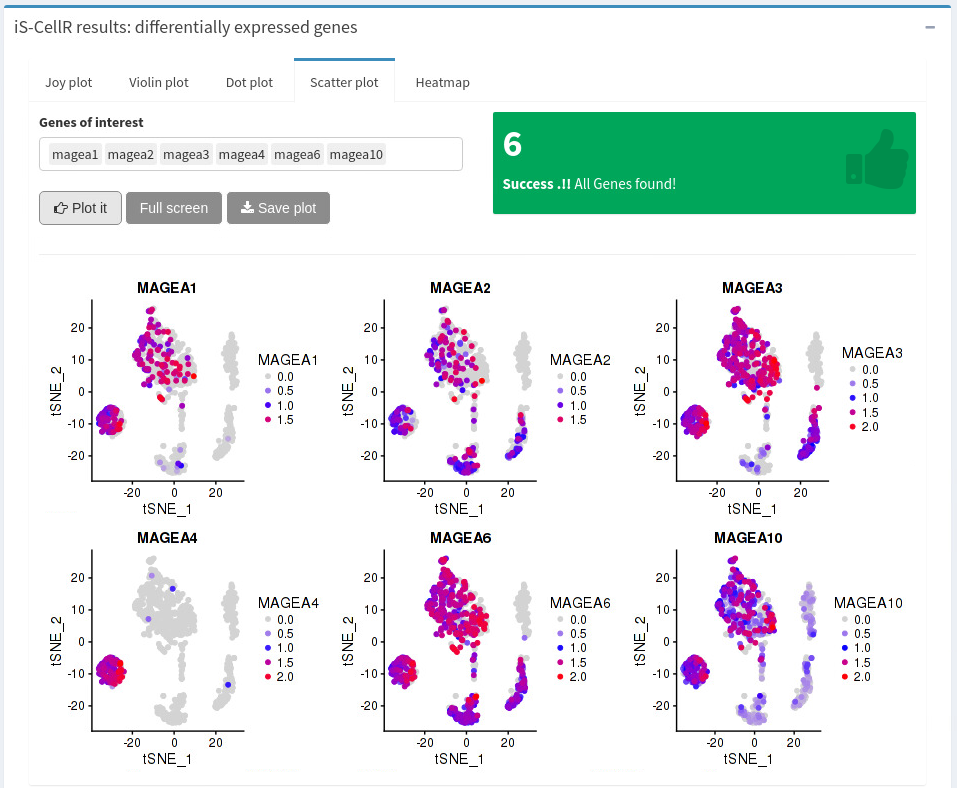


**Fig. S8D. Differentially expressed genes – Scatter plot.** The colours indicate the expression of genes from low to high expression, as light grey to red, respectively.


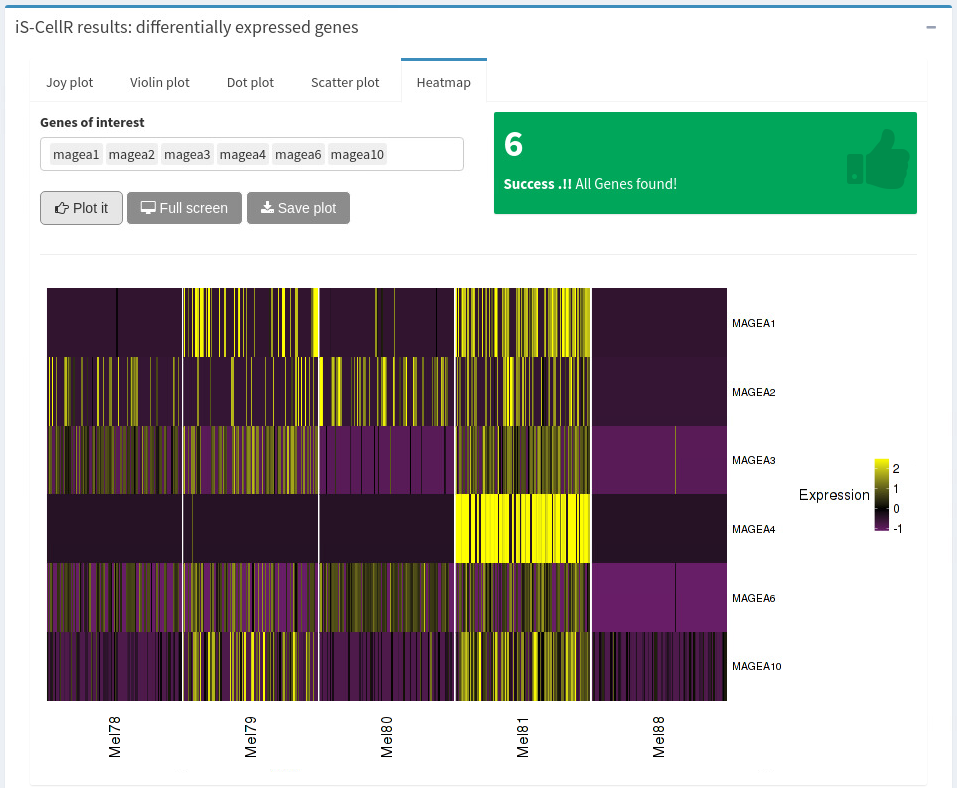


**Fig. S8E. Differentially expressed genes - Heatmap.**

**iS-CellR: visualising co-expression of two genes, study inter-/intra-sample heterogeneity**

The panel for discriminating marker genes contains three tabs – one for visualizing co-expression of two genes simultaneously, and one each for inter-sample and intra-sample heterogeneity. The first tab allows the user to plot expression levels of two genes simultaneously, together with the expression threshold (Figure S9A). It also displays the relative expression levels (minimum, maximum and mean) of both genes, which is very useful when applying an expression threshold. Gene expression levels above the threshold will be considered high, and levels below the threshold will be considered low, for each individual gene. When expression levels of both genes are above the threshold, they will be considered as high for both. The plot will be generated based on tSNE values and offers the same interactive features as the PCA plot. The user can study inter-sample heterogeneity based on gene expression levels of two sets of genes. A file containing a comma/semicolon/tab separated list of genes in a two-column format is required as an input. For inter-sample heterogeneity, the scatter plot will be generated based on the average expression values of GeneSet1 (x-axis) and GeneSet2 (y-axis) from all cells for each sample (Figure S9B). Similarly, for intra-sample heterogeneity, individual scatter plots will be generated using the average expression of GeneSet1 (x-axis) and GeneSet2 (y-axis) for each individual cell within each sample (Figure S9C).


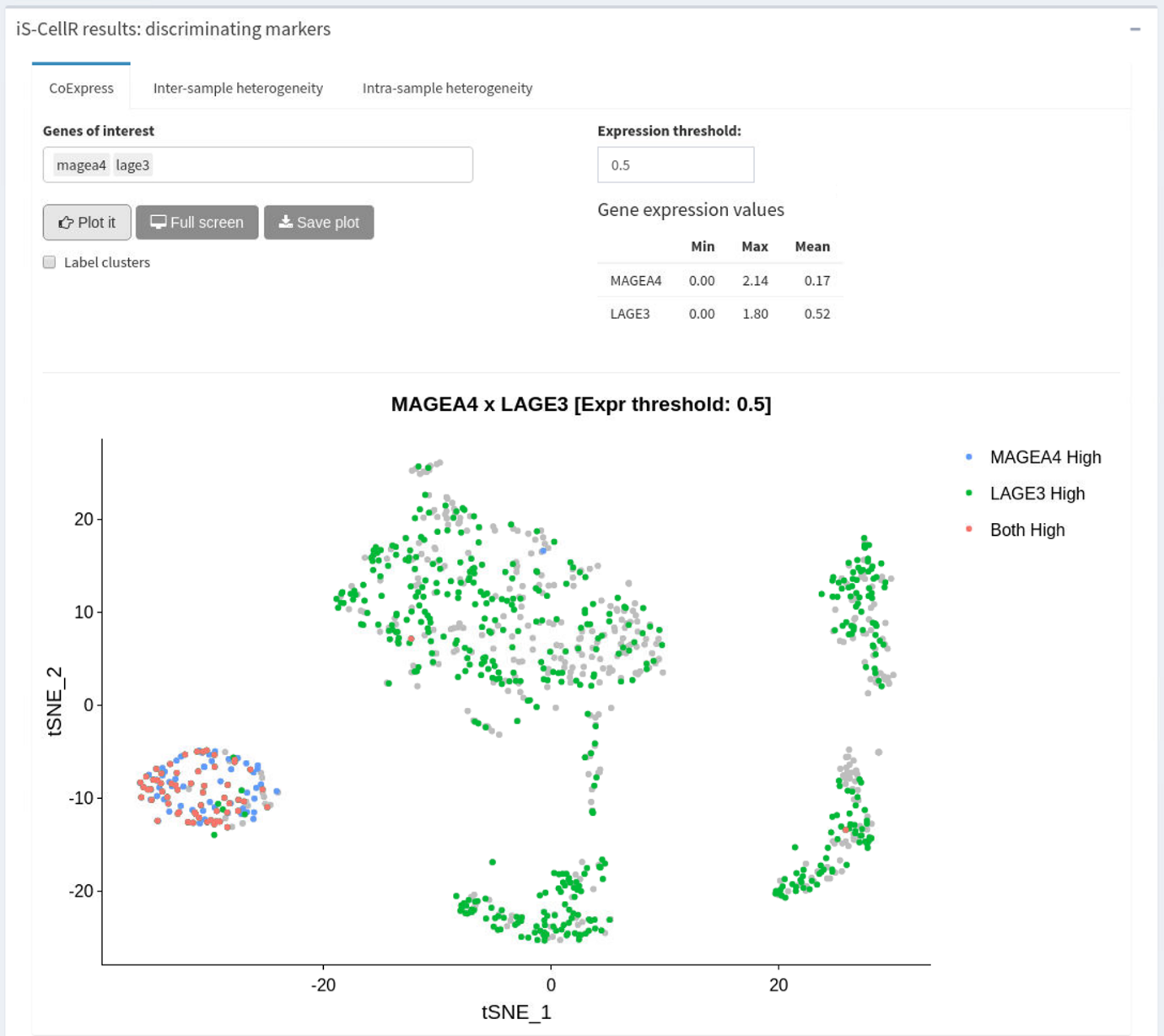


**Fig. S9A. Visualization of co-expression of two genes.** Co-expression of two genes can be visualized simultaneously on the same plot. The tSNE plot shows co-expression of genes MAGEA4 and LAGE3 with the expression threshold 1, where each point represents a cell and is coloured as red (expression >1 for both genes), green (expression >1 for LAGE3), blue (expression >1 for MAGEA4) and grey (expression = 0 for both genes).


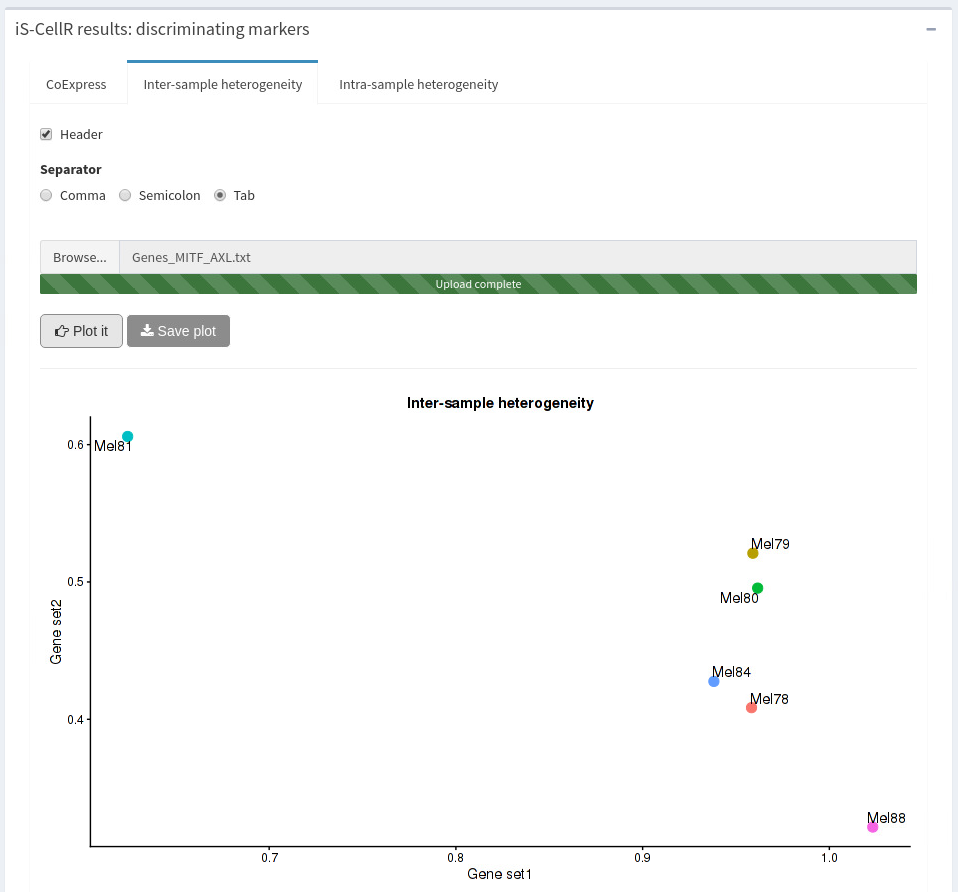


**Fig. S9B. Inter-sample heterogeneity.** The scatter plot shows the inter-tumour heterogeneity across six tumours based on the average expression of GeneSet1 (MITF) and GeneSet2 (AXL) in each tumour type. Both gene sets are MITF and AXL programme genes, as described by Tirosh *et al.,* 2016.


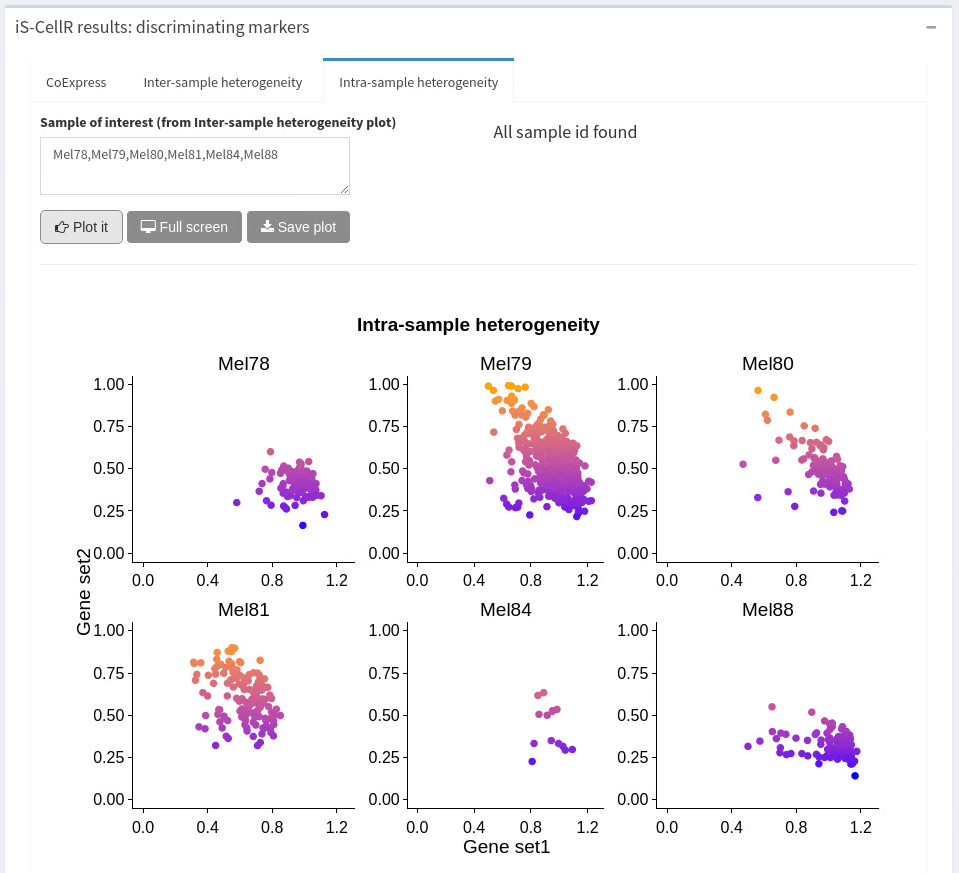


**Fig. S9C. Intra-sample heterogeneity.** The multiple scatter plots show the intra-tumour heterogeneity for each tumour based on average expression of GeneSet1 (MITF) and GeneSet2 (AXL) in each cell type. Both gene sets are MITF and AXL programme genes, as described by Tirosh *et al.,* 2016.

**Reference**

Tirosh,I. *et al*. Dissecting the multicellular ecosystem of metastatic melanoma by single-cell RNA-seq. *Science,* 2016;352:189-196.
